# Cathepsins and Their Endogenous Inhibitors in Host Defense During *Mycobacterium tuberculosis* and HIV Infection

**DOI:** 10.3389/fimmu.2021.726984

**Published:** 2021-08-04

**Authors:** Elsa Anes, José Miguel Azevedo-Pereira, David Pires

**Affiliations:** Host-Pathogen Interactions Unit, Research Institute for Medicines, iMed-ULisboa, Faculty of Pharmacy, Universidade de Lisboa, Lisboa, Portugal

**Keywords:** cathepsins, cystatins, serpins, tuberculosis, HIV-co-infection, host directed therapies

## Abstract

The moment a very old bacterial pathogen met a young virus from the 80’s defined the beginning of a tragic syndemic for humanity. Such is the case for the causative agent of tuberculosis and the human immunodeficiency virus (HIV). Syndemic is by definition a convergence of more than one disease resulting in magnification of their burden. Both pathogens work synergistically contributing to speed up the replication of each other. *Mycobacterium tuberculosis* (Mtb) and HIV infections are in the 21st century among the leaders of morbidity and mortality of humankind. There is an urgent need for development of new approaches for prevention, better diagnosis, and new therapies for both infections. Moreover, these approaches should consider Mtb and HIV as a co-infection, rather than just as separate problems, to prevent further aggravation of the HIV-TB syndemic. Both pathogens manipulate the host immune responses to establish chronic infections in intracellular niches of their host cells. This includes manipulation of host relevant antimicrobial proteases such as cathepsins or their endogenous inhibitors. Here we discuss recent understanding on how Mtb and HIV interact with cathepsins and their inhibitors in their multifactorial functions during the pathogenesis of both infections. Particularly we will address the role on pathogen transmission, during establishment of intracellular chronic niches and in granuloma clinical outcome and tuberculosis diagnosis. This area of research will open new avenues for the design of innovative therapies and diagnostic interventions so urgently needed to fight this threat to humanity.

## Introduction

Tuberculosis (TB), is an old infectious disease that has afflicted humans for about 70,000 years. It is mainly caused by *Mycobacterium tuberculosis* a species that colonized the world accompanying the Out-of-Africa migrations of *Homo sapiens* (1, 2). Comparatively, the human immunodeficiency virus (HIV) is a recent pathogen, transmitted to humans from primates in the 20th century with the first cases of acquired immune deficiency syndrome (AIDS) revealed after 1980 (3). TB was considered one of the great bacterial terrors of humankind, killing one in each seven people until the beginning of the 20th century with an impact similar to the black plague, being denominated likewise the ‘‘white plague’’.

With the development of the vaccine BCG in 1921 and the discovery of antibiotics, TB became finally a controlled disease. However in 1993, 50 years after the introduction of the first anti-TB drugs, the World Health Organization declared TB as a global emergency (4). The number of cases in Europe and North America, in addition to developing countries, were rising dramatically after decades of infection decline concomitant with the appearance of the first cases of multidrug resistant strains. The MDR-TB burden emerged with the human immunodeficiency virus (HIV) pandemic and and the majority of early MDR-TB outbreaks were observed in HIV-Mtb coinfected individuals in hospitals and prisons (5–7).

One quarter of the human population is latently infected with *Mycobacterium tuberculosis* (Mtb) (8). Latent TB (LTB) is defined as a state of persistent immune response induced by Mtb antigens without evidence of clinically manifested active TB (9) with absence of transmission between humans but with chest radiography often revealing the presence of lung granulomas that contain the bacteria. Co-infection with HIV completely alters this state, exacerbating the reactivation from latency to active TB. The progression to disease in humans is essential for the transmission and evolutionary survival of Mtb (10). Following a decade of AIDS, it was possible to observe the impact of HIV on the increase of TB cases and deaths (4).

Since 1996, HIV infection turned into a chronic illness, following the introduction of highly active antiretroviral therapies (ART) (11). Meanwhile, Mtb co-infection of HIV chronically infected people impacts progression to AIDS by exacerbating HIV replication, spread and genetic mutations (12). Actually, TB together with HIV infection remains in the 21th century the leading cause of mortality worldwide due to infectious agents (8).

The long and cumbersome TB treatment, consisting of a cocktail of four anti-TB drugs during a regimen of 6-9 months, together with patient non-compliance, contributed to the advent of multidrug resistant Mtb strains (MDR-TB) which renders first line antibiotics rifampicin and isoniazid ineffective (13). There are good epidemiological reasons to suspect that the HIV and MDR-TB pandemics are fueling each other. As stated, HIV infection render people more susceptible to develop active TB; HIV co-infection is a risk factor for contracting primary MDR-TB (14); HIV co-infected people with very low CD4^+^ lymphocyte counts (<100 cells/mm^3^ hallmark of advanced HIV infection), have been shown to be risk factors for developing resistance to rifampicin and to a lesser degree isoniazid (15, 16) and during co-infection low absorbance in the gut and low bioavailability of antibiotics (17, 18) provides sub-optimal concentrations that may contribute to MDR-TB and to the evolution of extensively drug resistant strains (XDR-TB). XDR-TB are supergerms with additional resistance to any fluoroquinolone and at least one of the three second-line injectable drugs (19). Over the past 40 years no new drugs were made available with the exception of bedaquiline and delamanid which were approved just for XDR-TB control (20, 21). Altogether these are threats to set back the progress against TB to the pre-antibiotic era.

Additionally, diagnosis of TB using sputum-based analysis, DNA methods or IFN−γ secretion (QuantiFERON, ELISpot) is problematic during HIV co-infection: a significant proportion of co-infected patients remain sputum smear-negative, hampering TB diagnosis (22). The restricted sensitivity and specificity of the traditional diagnostic methods are aggravated by the dismantling of TB granulomas during progression to AIDS and by the bacterial spread from lungs to other parts of the body, making chest X-ray analysis inconclusive (4, 23, 24). Moreover, higher rates of indeterminate results are found for patients with CD4 counts below 100 cells/μl, due to low levels of IFN-γ in response to stimulation (25). Gupta and coll. revealed in autopsy studies that TB remains undiagnosed at the time of death in approximately 50% of patients who are HIV-positive (26). Therefore, it is absolutely urgent to develop novel diagnostic and therapeutic tools for TB during HIV co-infection in tackling the global burden of these infectious diseases.

To respond to these challenges a deeper look at the particular host-pathogen interactions should define key targets of this arms race against Mtb and HIV. Both pathogens have to overcome mucosal barriers and access host cells where they establish chronic intracellular niches. In the last years we and others have been focusing on the study of host antimicrobial proteins namely cathepsins and their natural inhibitors cystatins and serpins as innate and adaptive immune responders against the infection but also revealing how both pathogens may subvert these responses to their own advantage (27–31).

Here we discuss recent advances on how Mtb and HIV interact with cathepsins and their inhibitors in their multifactorial functions during the pathogenesis of both infections. This area of research will open new avenues for the design of innovative therapies and diagnostic interventions so urgently needed to fight this threat to humanity.

## Cathepsins and Their Inhibitors

Metchnikoff, the father of innate immunity, first announced in 1901 that mononuclear phagocytes, which he named macrophages, take up exogenous material by phagocytosis into vacuoles containing the proteolytic enzyme macrocytase (32, 33). The term cathepsin, derived from the Greek word “kathepsein” was suggested later by Willstätter to describe the ability of a proteolytic enzyme “to digest” in a slightly acidic environment (34). Willstätter further proposed that cathepsin had its origin in leukocytes and was secreted after autolysis (34). These kind of proteases likely arose at the earliest stages of protein evolution in primitive organisms as simple enzymes necessary for complete protein digestion (35, 36). They evolved from simple proteases to a more sophisticated catabolism without complete destruction of proteins but rather generating new molecules retaining the properties and structure required for innate and adaptive immune responses (37).

Research on cathepsins is relatively recent, and mostly concomitant with the discovery of lysosomes by de Duve in 1955 (38). Lysosomes were associated with the major degradative vacuoles in eukaryotic cells, concentrating high amounts of cathepsins and providing a reducing and slightly acidic environment, optimal for their enzymatic activity.

In 1970, an enzyme was isolated from swine leukocytes, named cathepsin G due to its ability to digest gamma-globulin, followed by the isolation of cathepsin F, which hydrolysed fibrin (39). Nowadays, the term cathepsins describes proteases which are divided into three different families based on their catalytic mechanisms. These include the initial lysosomal enzymes, a group of 11 cysteine cathepsins [B, C, F, L, K, S, H, O, V (L2), W and X (P, Y, Z)], aspartic proteases (cathepsins D and E) and the serine proteases A and G (http://www.merops. sanger.ac.uk) (33, 37).

While initially considered as lysosomal proteases, with essential bulk proteolysis of endogenous and exogenous proteins, including direct digestion and killing of internalised pathogens, they are now known to act in diverse contexts of the cell. This includes (i) the cytoplasm, where they control cell death, autophagy (40) and cytokine trafficking, processing and turn-over (41), (ii) the nucleus where they control cell cycle and transcription (42) and, (iii) the plasma membrane, activating and shedding surface receptors and T-cell priming and signalling (28, 43). In host response to infections cathepsins play significant roles in innate as well as in adaptive immune responses. Cathepsin B is described to process and vesicle traffic immune modulators such as interleukin-1β (IL-1β) (41) and TNF-α (44), respectively; cathepsin K activates toll-like receptor 9 (TLR9) (45) and cathepsin S is involved in major histocompatibility complex (MHC) class II-dependent CD4^+^ T-cell activation (28, 43). Moreover they can be secreted out of the cell and have effects on extracellular matrix remodelling, mucosae host defence, angiogenesis, complement activation, as well as cellular processes including migration and proliferation (46). Recent studies have revealed that the diversity of protease substrates and biological effects triggered by their processing is far vaster than initially assumed. In non-lysosomal reducing non-acidic compartments other factors may contribute to cathepsin stabilization and activity. A good example is the glycosaminoglycans (GAGs) that can stabilize cathepsins at a neutral pH in the extracellular environment (47).

Importantly, protease activity requires tight regulation, and disruption of the close interplay between proteases, substrates and inhibitors may contribute to the pathogenesis and progression of a variety of diseases, including infectious diseases such as TB and AIDS.

The reversible endogenous inhibitors of cathepsins, which are their major regulators, can also be classified into different groups according to their mechanism of inhibition, namely serpins and cystatins (35, 37, 48). Serpins (serine protease inhibitors), block the active site of their target proteases through binding in a virtually substrate-like manner. By contrast, exosite-binding inhibitors like cystatins bind a region adjacent to the active site, thereby preventing substrate access to this site (36).

The cystatins (Csts) are the best characterized inhibitors and are further classified into four families: stefins including cystatins A and B are mainly found in the cytosol and the nucleus (Family I), extracellular and/or transcellular cystatins (Family II) EM, D, S, SA and SN are secreted and work as extracellular proteins (49, 50). Some secreted type II cystatins such as CstC and F can be internalized by immune cells or translocated from the secretory pathway accumulating in endosomal/lysosomal vesicles (51, 52). Type III cystatin family members include kininogens circulating in the blood as precursors of the vasoactive peptide kinin [cystatin families reviewed in (49, 50)]. Kininogens are also found in high concentrations in urine, sperm and lungs (53). Thyropins were described as family IV with the discovery that a fragment of the p41 invariant chain (p41Ii) associated with MHC class-II molecules inhibits cathepsin L (54).

## Cathepsins and Inhibitors During Mucosae Transmission of Mtb and HIV

**Figure 1:**
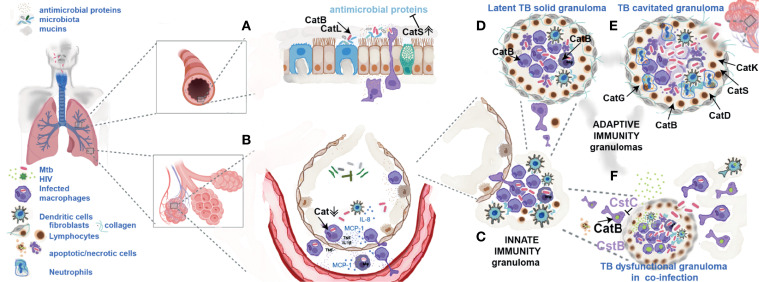
Cathepsins and their endogenous inhibitors during TB infection and HIV-co-infection. **(A)** Mtb has to overcome the strong mucosae inflammatory environment of the upper and lower respiratory tract where cathepsins B, L and S may have a direct killing effect. In inflammatory conditions their exacerbated secretion may contribute to cleavage of other antimicrobial proteins helping pathogen protection and transmission. **(B)** In the alveolar environment Mtb internalized by alveolar macrophages downregulates cathepsins contributing for establishment of intracellular niches. **(C)** Infected cells will move to the deeper lung tissue forming an innate granuloma. **(D)** Adaptive immune granuloma is a dynamic structure of apoptosis of infected macrophages induced by cytosolic cathepsins (as cathepsin B) releasing live bacilli in apoptotic bodies that are readily internalized by new arrival macrophages. **(E)** During active TB, the exacerbated secretion of cathepsins G, K, D, S and B in the granuloma and from cells in the lung tissue stimulated by Mtb products leads to collagen destruction, cavitation and, bacteria spread to other humans. **(F)** HIV arrival to the lung in latently infected macrophages, under an inflammatory environment of cytokines will start to replicate. Cathepsin B by processing viral proteins will participate in the formation of new viral particles. Csts B and C will inhibit this process. Co-infection with HIV leading to lymphocyte depletion, death of monocytes at the site of virus replication and low secretion of TNF compromises the dynamics of granuloma, their integrity, culminating with infected cells spread to other parts of the body.

**Figure 2:**
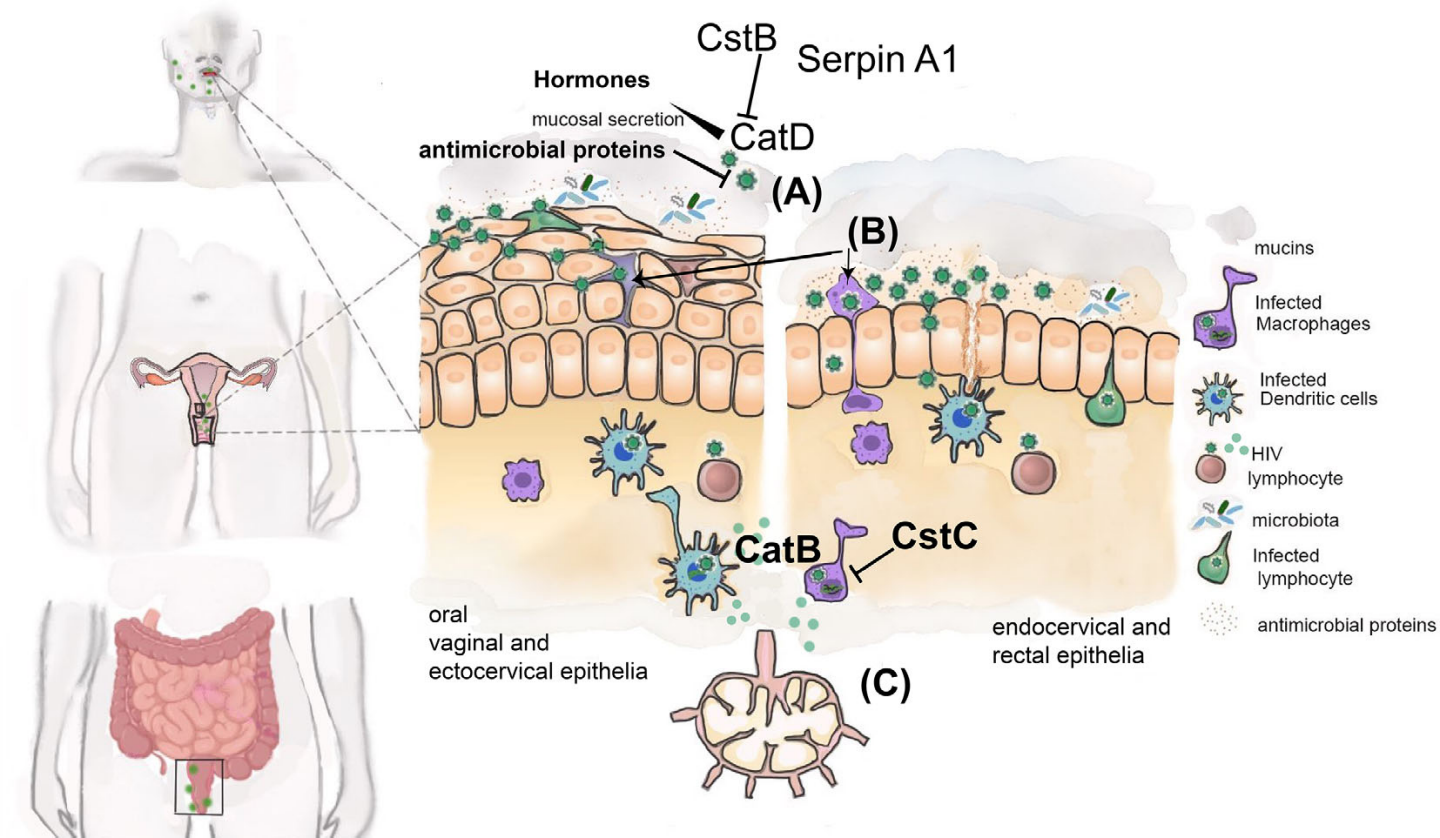
Cathepsins and their endogenous inhibitors during sexual transmission of HIV. Schematic representation of different mucosae involved in sexual transmission of HIV. HIV may reach the mucosae as free viral particles **(A)** or carried by latently infected cells (e.g. T-CD4+ lymphocytes and macrophages) **(B)** able to transmigrate between epithelial cells. HIV must traverse the epithelial cell layer and gain access to immune cells present in subepithelial layer. The mucus layer enriched with several antimicrobial proteins secreted in response to microbiota and pathogen MAMPs further contributes to inactivate HIV. The presence of cathepsin D (CatD) secreted by vaginal epithelia can facilitate HIV transmission by enhancing its ability to infect CD4+ T-lymphocytes and epithelial CD4-negative cells, providing an additional mechanism used by HIV to reach subepithelial layer. Regulation of cathepsin D by hormones as well as semen and local microbiota influence the infectivity of HIV. Cathepsin inhibitors Cst B, Serpin A1, Serpin A3, correlates with protection from HIV transmission. Infected DCs and macrophages may amplify virus replication by processing viral proteins using cathepsin B **(C)**. Cst C will inhibit this process. HIV is then able to infect susceptible target cells (CD4+ T-lymphocytes) either directly or after viral transfer from DC or macrophages at regional lymph nodes.

An important step in the course of Mtb and HIV infections is the initial contact of Mtb and HIV with epithelial cells and cells patrolling mucosae as is the case of dendritic cells (DCs) and macrophages. Both pathogens must overcome mucosal barriers to establish chronic intracellular niches in their host cells. Mtb is transmitted through small droplets that need to overcome the potent innate immune antimicrobial milieu of the upper and lower respiratory tract to reach the more sterile alveolar compartment where macrophages and DCs internalize the bacteria by phagocytosis (**Figure 1**) (10). HIV transmission occurs mainly through genital and anal mucosae. The two types of HIV (HIV-1 and HIV-2) are able to infect cells expressing the CD4 receptor and a chemokine coreceptor (usually CCR5 or CXCR4). The sequential interaction of viral envelope (gpSU) with CD4 and coreceptor molecule ultimately leads to the direct fusion between viral envelope and plasma membrane (55) with subsequent release of viral capsid into cell cytoplasm. According to the referred requirements of cell membrane receptors, and depending on the environment, HIV may infect epithelial CD4-negative cells, macrophages, dendritic cells and resident CD4^+^ T-lymphocytes (**Figure 2**) (56–59).

Mucosal barriers possess intrinsic microbicidal properties mostly based on innate receptors such as toll like receptors (TLRs). These sense microbial signatures from pathogens and microbiota (MAMPs) leading to the secretion of antimicrobial proteins and peptides. Among these secreted proteases are cathepsins, the cell wall-degrading enzyme lysozyme, the iron-chelating protein lactoferrin, the secretory leucoprotease inhibitor (SLPI) and specific membrane-permeabilizing members of the defensin, cathelicidin, and pentraxin families of antimicrobial peptides (AMPs) [reviewed in (60)].

There are a myriad of other secreted proteins that are specific of the lungs including the surfactant proteins SP-A and SP-D from the complement system (61). They belong to collectins, a group of related proteins important for opsonizing bacterial pathogens and to modulate the surface tension in alveoli during breathing. SPs A and D play a role in surfactant homeostasis and host’s defence in the lung by their involvement in the early capture of Mtb. They bind to the terminal mannosyl oligosaccharides of the cell wall which induces bacilli agglutination and thus improves phagocytosis by alveolar macrophages (61).

In the lung environment the two prevalent endopeptidase cathepsins B and L, are primarily found in bronchial epithelial cells, which contribute to the first line of defence against pathogens. Cathepsin S is expressed mainly in macrophages and is present also on the surface of ciliated cells and may favour the motility of cilia by preventing unspecific binding with circulating plasma-derived proteins (62).

Lactoferrin found in all mucosae has been shown to be active against a number of viruses including HIV (63). SLPI expressed by macrophages, neutrophils and the mucosal surface of epithelial cells is found in large quantities in bronchial, cervical, and nasal mucosa, saliva, and seminal fluids. Studies have shown that decreasing levels of SLPI in saliva also decreases its anti-HIV activity (64).

AMPs such as cathelicidins have been shown to be determinants of resistance to Mtb (65). The human hCAP-18/LL37 active peptide LL-37 is the only member of the cathelicidin family identified to be expressed in human aerial epithelial cells and alveolar macrophages, and it is a major antimicrobial peptide in the innate immune response against Mtb (65). Human *β*-defensin-2 (HBD-2) is another known inducible antimicrobial peptide associated with the pathogenesis of human TB that has the capacity to control the growth and chemotactic activity of Mtb (66).

The appropriate function of innate immune responses in the mucosae to control pathogens depends on the equilibrium and function of proteases and their inhibitors. Some proteins may work as antimicrobials in one context and as inhibitors of proteases in other inflammatory environments, thus compromising the antimicrobial activities of such blocked proteases. For instance, SLPI works either as antimicrobial protein and as an inhibitor which protects epithelial tissues from serine proteases including cathepsin G and neutrophil elastase while preventing their potent antimicrobial activity (67, 68). Cathepsins B, L, and S have been shown to cleave and inactivate lactoferrin, SLPI and AMPs including defensins and cathelicidins (**Figure 1**) (60, 69). The surfactant proteins A and D were identified as potent substrates of cathepsins B, L and S, which may impair their capacity to aggregate lipids and bind microorganisms (53). AMPs are somewhat resistant to proteolytic degradation, however, cathepsin S (**Table 1**), is the most potent cysteine cathepsin able to cleave all the AMPs mentioned and impair their antimicrobial activity. In chronic lung diseases associated with inflammation, overexpression of cathepsin S and other cathepsins may lead to increased degradation of AMPs such as defensins HBD-2 and -3, thereby favouring Mtb infection and colonization (69).

**Table 1:** Resume on cathepsins and protease inhibitors role in Mtb and HIV infection.

Protease/protease inhibitor (expression)	Compartment	Target	Effect	Ref
**Cathepsins B, L and S (increased)**	Upper/lower respiratory tract mucosa	Inactivate LF, SLP1, AMPs, SP-A and D	Favors Mtb colonization	(60, 69, 70)
**Cathepsin D (increased)**	Genital mucosa	Enhance infection of CD4+ and epithelial cells by Increasing HIV Env glycoprotein affinity for CD4 receptor	Favors genital transmission of HIV	(71, 72)
**Cystatin B**	Genital mucosa	Inhibition of cathepsin D	Resistance to genital transmission of HIV	(73)
**Serpins A1 and A3**	Genital mucosa	Inhibition of cathepsin G	Resistance to genital transmission of HIV	(73)
		Prevents HIV binding to cell receptors		
**Lysosomal cathepsins**	Endosomal pathway of host cells	Direct killing of the pathogen	Mtb and HIV down regulate cathepsins helping intracellular niche establishment	(27, 57)
		Antigen processing and presentation		
**Cathepsin B**	Endosomal pathway of host cells	Release of HIV-Gag particles during assembly and maturation in macrophages	Increases transmission of HIV to neighbor cells of new released viral particles	(31)
**Cystatin B**	Cytosol	Interference with interferon β response	Induces HIV replication and transmission	(74)
**Cystatin C**	Endosome	HIV protease inhibition	Prevents maturation of viral particles and HIV infection of new cells	(75)
	Cytosol			
**Lysosomal cathepsins B, H, K, L, S, D (increased)**	Cytosolic release	Apoptosis	Adaptive Mtb granuloma	(35, 44)
**Cathepsin B**	Cytosolic release	Inflammasome activation	Granuloma cavitation in active Mtb	(76–78)
		Pyronecrosis	Transmission to new host	
**Cathepsin G**	Macrophage membrane	Binding to extracellular Mtb	Mtb prevents cathepsin G proteolytic activity	(79, 80)
			Extracellular Mtb growth	
**Cathepsins G, B, K, S, V, D**	Extracellular	Extracellular matrix destruction	Lung parenchyma destruction	(53)
			Inflammation in TB granuloma cavitation	
			Mtb transmission to new host	

LF, Lactoferrin; SLP1, secretory leucoprotease inhibitor; AMPs, antimicrobial peptides; SP-A and D, surfactant proteins A and D.

By contrast, certain AMPs may also work as inhibitors of proteases (53) supporting a regulatory role in lung innate immunity. This is the case of the cathelicidin hCAP-18 N-terminal prodomain, also called cathelin domain (CLD) because it shares a common structural fold with chicken cystatin, and was proposed to inhibit the activity of cathepsin L (81).

Therefore, pathologic determinants leading to increased secretion of cathepsins may account for pathogen transmission. This is the case of chronic lung diseases such as cystic fibrosis and chronic obstructive pulmonary disease (COPD) or tobacco smoking (82). Cysteine cathepsins are also up-regulated during human papillomaviruses HPV16-induced cervical carcinogenesis and other tumours (83, 84).

Cleavage of innate immune proteins results in loss of their antimicrobial effects. In the case of cathepsin S, due to its ability to exert elastase activity, to inactivate airway host defence proteins and AMPs, its capacity to induce ECM remodelling and to alter mucus production across a wide pH range, is a strong contributor for pathogen transmission in extracellular environment (70). Cathepsin S release is regulated by several pro-inflammatory cytokines, such as IL-1β and TNF-α that respond to MAMPs sensing by innate receptors (61).

Extracellular cathepsins are particularly important in directly modulating the immune environment at lower or upper female genital tract (FGT) and not surprisingly also affecting HIV transmission (**Figure 2**, **Table 1**). It was demonstrated that pretreatment of HIV strains with cathepsin D not only enhanced infection of CD4+ T-lymphocytes but also allowed infection of epithelial CD4-negative cells (71). Interestingly, similar enhancement was observed when viruses were pretreated with vaginal secretions (71, 72), or human milk (85). In both cases the contribution of cathepsin D could be demonstrated. This expanded tropism in the presence of vaginal secretions has obvious implications on HIV genital transmission as it may allow infection of the epithelial cell layer present in sexual mucosae. The mechanism of cathepsin D enhancement may rely on proteolysis of HIV viral envelope glycoprotein gpSU modifying its structure and thus increasing the affinity of gpSU-CD4 receptor interaction. Apparently, this may even allow viral entry into cells without prior interaction with CD4 receptor.

The regulation of cathepsin D by hormones involved in menstrual cycle (86), together with the described interference of cathepsins with several anti-HIV innate immune factors (63, 64, 69, 87, 88), provide an extra level of complexity to the regulation of factors affecting HIV ability to infect cells at the genital mucosae.

Accordingly, the cathepsin inhibitors Cst B, Serpin A1, Serpin A3, found to be significantly over-abundant in cervicovaginal fluid of HIV resistant women, indeed correlate with protection from HIV transmission through unprotected sexual intercourse (73).

## Cathepsins and Inhibitors in the Establishment of Chronic Intracellular Niches and Immune Responses

*Mycobacterium tuberculosis* and HIV are both intracellular pathogens. Mtb express a surface lipid phthiocerol dimycocerosate (PDIM) that masks the MAMPs so that they are poorly detected by the host innate immune system during their passage in the upper and lower respiratory tract (10). Concomitantly, they use a related surface lipid, phenolic glycolipid (PGL), to induce the macrophage chemokine CCL2 (also named as monocyte chemotactic protein 1 or MCP-1) to recruit macrophages that are Mtb growth-permissive after phagocytosis in the alveoli environment (**Figure 1**).

For HIV, it is thought that the virus primarily invades the patient by infecting resident macrophages, dendritic cells, activated CD4^+^ T lymphocytes, or mucosal CD4-negative epithelial cells in the vaginal, cervical or rectal mucosae. In the early days after transmission, it is postulated that the virus replicates to high levels in macrophages in regional lymph nodes (89). HIV infection of macrophages is a major milestone in viral pathogenesis. Since these cells constitute one of the reservoirs where HIV could be maintained in a stable latent state, they persist infected even after antiretroviral therapy and constitute a major obstacle to eradication of HIV infection (56). In addition, the infection of macrophages provides an important advantage for viral dissemination to CD4^+^ T lymphocytes, exploiting the cell-to-cell transmission through virological synapses [reviewed in (90)].

The virus uses two major mechanisms to enter cells: direct fusion with the cytoplasmic membrane with subsequent release of viral capsid into the cytosol (91) and the endocytic pathway followed by fusion with the endosome membrane (92).

Any pathogen that is phagocytosed/endocytosed by cells is meant to be destroyed in the endolysosomal pathway after contact with activated proteases, such as cathepsins, in an acidic environment. Thus, intracellular pathogens have evolved diverse ingenious signature strategies to exploit or circumvent the hostile endocytic milieu. In line with this, by blocking the acidification of lysosomal compartments, HIV increases its infectivity allowing the virus to access the cytoplasm (93–95). Mtb is reportedly found localized to non-acidified early endosomes following Mtb-induced blockage of phagolysosome maturation (96) or found in acidified lysosomes (97) with a small proportion of the bacteria eventually breaking out of the phagosome to reside in the cytosol (98).

Regardless which kind of cell is infected, HIV (as all retroviruses) is able to retrotranscribe its RNA genome to a double stranded DNA (dsDNA) molecule and to integrate it into the host cell chromosome. The integrated genome (referred as provirus) could establish a reversible latent state, lasting the lifespan of the infected cell, or serve as template to both messenger RNAs and to viral genomic RNA (99). The latter will be encapsidated and new virions are released by budding at the plasma membrane. Finally, viral maturation, consisting on proteolytical processing of capsid proteins by a viral encoded protease, occurs after egress of immature viral particles (100).

The control of proteases’ activity may account for the success of the pathogen in establishing chronic intracellular niches or otherwise be completely digested and cleared from cells.

### During HIV Infection

Cathepsin B has been associated with HIV replication in macrophages by facilitating the release of HIV-Gag particles during assembly and maturation of viral particles. The blockade of cathepsin B by a specific inhibitor (CA-074Me) or the usage of cathepsin B knockdown cells impaired the release of HIV-Gag which accumulated in compartments sharing the same markers of multivesicular bodies and autophagosomes (31).

HIV replication in macrophages is also influenced by the presence of Cst B, an inhibitor of cathepsins B, L and S. Interestingly, the role of Cst B in HIV replication depends on its localization: intracellular Cst B induces HIV replication on macrophages derived from peripheral blood monocytes (74, 101), whereas the overexpression of Cst B in cervicovaginal fluid correlates with protection from HIV transmission through unprotected sexual intercourse (**Table 1**) (73).

Furthermore, HIV infection of macrophages seems to induce an up-regulation of both intracellular and secreted Cst B (102, 103). This would influence several HIV-host interactions with divergent actions depending on cellular and tissue localization. For example, intracellular Cst B inhibits the interferon beta response by blocking signal transducer and activator of transcription-1 (STAT-1) entry into the nucleus and by simultaneously reducing the levels of tyrosine phosphorylated STAT-1 (STAT-1PY) (74). While STAT-1 induces HIV replication, STAT-1PY is associated with inhibition of HIV replication (104). The tissue where macrophages reside is also an important factor regarding the levels of extracellular Cst B. The analysis of placental macrophages’ (PM) secretome revealed a lower Cst B concentration when compared to peripheral blood monocyte-derived macrophages (MDM) (101, 103). Since downregulation of Cst B by siRNA silencing reduced the replication of HIV in MDM, it is appealing to associate the lower amounts of Cst B in PM secretome with an innate protection against maternal-foetal HIV transmission (101). Further studies may provide a clarification of this association and may lead to the confirmation of a potential new target for HIV restriction in MDM, one of the main reservoirs of HIV.

As referred earlier, HIV infection is mainly a mucosal infection and dendritic cells (DCs) are one of the first cells HIV encounters to overcome the barrier that lines genital tract mucosae. In DCs, the fate of HIV relies heavily on its capacity to evade immune sensing while it infects, persists, or replicates inside DCs. HIV has evolved several strategies that enable it to preclude pattern recognition receptors (PRRs) sensing of viral components, particularly nucleic acids (105). One such strategy of paramount importance is related with high levels of DC-specific intercellular adhesion molecule-3 grabbing non-integrin (DC-SIGN) expression and low levels of the viral receptor CD4 at DCs’ membrane. Binding to DC-SIGN prevents HIV from entry in DCs through a pathway that supports productive infection: the viral envelope fusion mediated by CD4/coreceptor CCR5 or CD4/coreceptor CXCR4 interactions. Instead, HIV is targeted to endocytic vesicles where the majority of internalized viral particles will be degraded through cathepsin-mediated proteolysis in late endosomes, while the remaining viruses (roughly 5-10% of the initial inoculum) are redirected to DC/T-lymphocyte immunological synapse (106–108). Regardless of the pathway, neither are capable of triggering PRR sensing and subsequently induce full DC maturation, a requisite for appropriate priming of T-lymphocytes. So, although DCs are able to present HIV-derived antigens to T-lymphocytes, they show a reduced capacity to mature and thus to trigger an adequate immune response.

In line with this mechanism, HIV counteracts another pathway that otherwise would lead to its lysosome-mediated total degradation: during *de novo* infection, HIV is able to markedly decrease the expression of lysosomal cathepsins B, C, S and X (previously named Z), as well as Cst C (57). This downregulation seems to occur 48 h after infection of monocyte-derived DCs by replicative HIV-1BaL, an HIV-1 strain able to productively infect DCs (109). Conversely, exposure to an inactivated version of HIV-1BaL, fails to show any alteration on the expression of the referred cathepsins/cystatins (57). These in turn enhance transfer of infectious virus to T-lymphocytes across the immunological synapse. Finally, and since cathepsins are directly involved in viral antigen processing, it is expected that the reduction of their expression leads to a less than optimal activation of the CD4^+^ T-lymphocytes (57).

Additionally to the canonical pathway of viral entry, involving the sequential engagement of CD4 and a coreceptor (usually CCR5 or CXCR4) referred above, some HIV strains are able to infect CD4-negative cells through a mechanism involving the direct interaction of envelope gpSU with the coreceptor molecule. This mechanism has been mostly referred in HIV-2 and less frequently in HIV-1 (110–113). This CD4-independent entry not only implies that coreceptor binding site is already exposed and susceptible to neutralization by antibodies targeting this crucial envelope region, but also seems to impose the endocytosis of HIV virions and their potential exposure to acidic late endosome/lysosome degradation by cathepsins [reviewed in (59)]. Accordingly, it was demonstrated that either Cst C, which inhibits cathepsin B, or CA-074Me, could enhance CD4-independent infection by HIV-1 (58). This indicates that cathepsin B activity reduces the susceptibility of target cells to CD4-independent infection while not affecting CD4-dependent infection. In contrast, HIV entry through a CD4-dependent mechanism occurs either by direct fusion between viral envelope and plasma membrane or by envelope fusion with endosome membrane. By using this entry process, HIV hides the coreceptor binding site until the late entry stage and provides an alternative to the endocytic pathway and the potential degradation by cellular proteases.

Cst C is expressed predominantly in the male reproductive tract and is abundantly present in semen. As stated before, this inhibitor may be internalized and concentrated in the endocytic pathway. Experiments *in vitro* indicate that Cst C binds to various HIV proteins including HIV protease. The function of HIV protease is to cleave newly synthesized proteins at the appropriate places to create mature functional protein components of an infectious HIV virion. It was suggested that Cst C may abrogate the action of HIV protease, compromising the normal functioning of viral protease which in turn would potentially prevent viral replication and transmission (**Table 1**) (75).

### During Mtb Infection

A large part of the research on Mtb infection of their major host cells, the macrophages, was made using non-human cells with mycobacteria species other than *Mycobacterium tuberculosis.* Mtb is a strict human pathogen. Other mycobacteria species such as the attenuated *M. bovis* vaccine strain BCG or *M. avium*, which is not a particularly successful human pathogen, lack the major region of difference 1 (RD-1), coding for relevant virulence determinants of Mtb (10, 114). Some of them as the secretory mycobacterial antigen ESAT-6/ESX1, CFP-10 and others such as ManLAM and PGL are involved in arresting phagosome maturation, lysosome permeabilization, escape to cytosol, inflammasome activation and in granuloma formation (10, 115–117). Therefore, in some of the published data limitations inherent to the model of macrophage and mycobacteria used should be taken into account. Nevertheless, Mtb and *M. avium* have been observed to possess similarities in blocking phagolysosome fusion and phagosome acidification, and are thus capable of growing/survive within these intracellular niches (118).

Schaible and coll (119) early studies on mouse bone marrow macrophages described the acquisition of procathepsin D from the biosynthetic pathway and their processed forms present in the same compartment of latex beads in the endocytic pathway. Phagosomes containing live *M. avium* were able to access the precursor of the cathepsin but not the matured forms while the mature active protease was found in the same vacuole as dead pre-internalized bacteria or latex beads. Using murine macrophages Singh et al. (120) showed that Mtb virulent strain H37RV was able to exclude vacuolar proton ATPase (v-ATPase) from phagosomes. This was shown to lead to poor acidification and weak activation of cathepsin D into the active form, concomitant with reduced processing of the Mtb immunodominant antigen Ag85-B.

Other studies using Mtb infection in mouse macrophages inferred that cathepsin D delivery was indeed dependent on the cell traffic important regulator GTPase Rab7 [for review on Rabs see (121)]. Rab7-acquisition by late endosome membrane is required for maturation of phagosomes into late endosomes/lysosomes (122, 123). Mtb internalization caused dissociation of Rab7 from the bacteria-containing phagosome leading to their entrapment in a non-matured vesicle (124). Recently, *M. tuberculosis* Rv0297PGRS domain was revealed to be a player in this game. The recombinant protein expressed in non-pathogenic mycobacteria induced low acquisition of cathepsin D and Rab7 in the human cell line THP1, by interference in the maturation of phagolysosomes (125).

Using microarray analysis, our group previously showed that mycobacterial inhibition of the proinflammatory transcription factor NF-κB results in impaired delivery of lysosomal enzymes to phagosomes and reduced *M. avium* killing (126). Thus mycobacteria manipulation of cell trafficking, and consequent avoidance of late endosome fusion and delivery of cathepsin D or other proteases, allows them to effectively survive intracellularly.

Furthermore, our group recently investigated the role of cathepsins and their inhibitors at the level of gene expression, gene regulation and enzymatic activity during Mtb infection of human primary macrophages (27–29). At the level of gene expression, Mtb induces a general downregulation of cathepsins mRNA either in resting/permissive M0 macrophages or in IFNγ-induced inflammatory M1 macrophages. In contrast, *M. smegmatis*, a non-pathogenic mycobacteria that is efficiently killed by macrophages (127) induces up-regulation of most cathepsins in both M0 and M1 type of cells. Moreover during Mtb infection most cystatins remained downregulated in M0 macrophages to allow full cathepsin proteolysis (27). We infer that this will not impact cathepsins activity as they are found downregulated too. A siRNA knockdown screen of a large set of cathepsins revealed that almost half of these enzymes have a role in pathogen killing, while only cathepsin F coincided with Mtb resilience (27).

Surprisingly, the analysis of the full-length cDNA of human cathepsin F predicts the presence of a cystatin domain at the N-terminus of the cysteine protease zymogen supporting the hypothesis that the cathepsin F gene resulted from a gene fusion between an ancestral cystatin and a cathepsin gene where its “cystatin-like” domain, might function as an endogenous cysteine protease inhibitor (128). Under this assumption and in line with our observation, cathepsin F may be acting as a cystatin, contributing towards increased Mtb survival. Additionally the pharmacological treatment with a general inhibitor of cysteine cathepsins E-64d, or with Cst C-based inhibition of cathepsins B, S, and L significantly impacts *M. tuberculosis* survival (27).

At the level of gene regulation Mtb indeed manipulates macrophage microbicidal functions through post-transcriptional control by microRNAs (miRNAs) [reviewed in (129)]. Functionally, miRNAs are a class of small noncoding nucleotides (18–25), endogenous RNA molecules that regulate the translation of messenger RNAs (mRNAs) causing their degradation or protein translation inhibition to maintain optimal levels of the target protein (130). Therefore microRNAs were investigated with potential involvement on cathepsins mRNA targeting during mycobacteria infection.

Curiously using algorithms tools such as the miRmap to predict miRNA targets more than one thousand are predicted to target cathepsin S with the top 30 including miR-106b-5p and miR3619-5p. Both miRNAs were shown to be manipulated during mycobacteria infection (28, 131). For miR-106b-5p, we found it to be strongly up-regulated during Mtb infection among several other microRNAs in opposition to what it is observed with the non-pathogen *M. smegmatis* (28). Concomitantly, cathepsin S downregulation not only led to enhanced Mtb survival but also led to poor MHC class II cell surface expression and poor priming of T-lymphocytes. Modulation of miR-106b-5p did not impact necrosis, apoptosis or autophagy arguing that miR-106b-5p directly targeted cathepsin S expression as a way for Mtb to avoid exposure to degradative enzymes in the endocytic pathway. Another study demonstrated the downregulation of miR-3619-5p by BCG in THP-1 macrophages, leading to up-regulation of cathepsin S with impact on autophagy (131).

Additionally, IL-10 dependent inhibition of cathepsin S by *M. bovis* has been observed (70, 132). Regulation of cathepsin S provides a mechanism by which mycobacteria can attenuate MHC class II surface expression and promote immune evasion (28). Moreover, the effects of IL-10 could be overcome using recombinant BCG expressing active cathepsin S (133).

At the level of enzymatic activity our recent studies indicated that the pharmacologic manipulation of cathepsins B, L and S may be achieved using saquinavir, a protease inhibitor used to control HIV infection (30, 134). Protease inhibitors, acting at post-integrational stages of virus replication cycle (135), are the only drugs able to interfere with virus production and release from macrophages during infection (136).

In the context of Mtb infection including co-infection with HIV our group demonstrated that saquinavir, in contrast to ritonavir, is able to induce a significant increase of endolysosomal proteases activity especially of cathepsin S. The enhancement of the catalytic activity was able to overcome the enzymatic inhibition induced by the pathogen in a three-fold magnitude. Therefore saquinavir may be repurposed as host directed therapy for TB (29). The treatment of infected macrophages led not only to an exacerbated intracellular killing of the bacteria but also to an improved expression of the HLA class II antigen presentation machinery at the cell surface, to CD4+ T-lymphocyte priming and proliferation as well as to increased secretion of IFN-*γ*.

## Cathepsins and Inhibitors in Granuloma Clinical Outcomes and Diagnostics

Permissive alveolar macrophages infected with Mtb then move to deep lung tissue and are at the centre of the granuloma formation a process that starts shortly after infection (2, 10, 137). These primary foci of infected cells will secrete cytokines as TNF-α and IL-1β and chemokines as MCP-1 and interleukin-8 (IL-8 or CXCL8) (138). The first leads to macrophage and endothelial activation and the latter to chemoattraction of a range of immune cells (additional macrophages, lymphocytes and DCs) (139). The expression at the cell surface of leukocytes of particular sets of chemokine receptors provides an “exclusive combinatorial *address code*” for the correct layer positioning within the granuloma (138). All together will form a stratified structure with the infected macrophages at the core (**Figure 1**). This host-pathogen interaction is dynamic and consists of an equilibrium between death of infected macrophages and replenishment through continuous cellular recruitment and *de novo* infection with bacteria from cellular debris (137).

This process leading to the death of infected cells and new phagocytic events enables a remarkable expansion of the bacterial niche, always within macrophages (10) and where cathepsins play a relevant role.

Several direct or indirect factors cause lysosomal damage with the release of cathepsins (B, H, K, L, and S) into the cytosol (**Table 1**). In turn these enzymes cleave Bid, a cytoplasmic factor that releases cytochrome C, activating apoptosis. Apoptosis is also triggered by the release of apoptosis inducing factor (AIF) by cathepsin-D-activated Bax [reviewed in (35)].

In infected macrophages cathepsin B is particularly related with acute inflammation and with cell death either by apoptosis or necrosis. Moreover, cathepsin B is involved in the posttranslational processing of TNF-α and its trafficking in vesicles to the macrophage plasma membrane. Secreted TNF-α potentially induces apoptosis of infected macrophages (44).

In addition, the coordination of macrophage death and re-phagocytosis is mediated through the Mtb virulence secretion system ESX-1, *via* its secreted effector ESAT6. ESAT6 is known to induce membrane pore formation, including in lysosomes, and is one of the known factors allowing cathepsins and Mtb effectors to reach the cytosol (76, 77, 117). Likewise, lysosomal induced apoptosis and necrotic cell death of infected cells in culture has been described by multiple ways with some of them functional in the granuloma.

The release of mature cathepsin B into the cytosol was recently demonstrated during Mtb infection of macrophages, resulting in inflammasome activation and secretion of pro-inflammatory cytokines such as IL-1β (76, 77). These events were concomitant with macrophage cell death depending on bacterial burden. In fact, cathepsin B mediates inflammasome activation under low bacterial burden allowing IL-1β release that stimulates prostaglandin-E2 (PGE2) secretion. PGE2 in turn protects macrophage from necrotic cell death while impacting apoptosis (77, 78). This cell death process culminates with bacteria enclosed within apoptotic vesicles that are readily internalized by recently recruited macrophages. This is likely the situation that often occurs during latent TB. In contrast, when macrophages are infected with high burdens of Mtb secreting ESAT-6, an exacerbated induction of inflammasomes in the absence of cathepsin B is responsible for high extension of necrotic cell death (76). This is what likely occurs in the necrotic TB granuloma.

Pathogenic mycobacteria may also influence the level of cathepsin G by direct binding inhibition in macrophages (79). The Mtb Rv3364c protein binds to cathepsin G on the host membranes, inhibiting its enzymatic activity and the downstream activation of caspase-1-dependent cell death. Moreover, in mice granulomas infected with Mtb, large amounts of the serine protease cathepsin G together with serine protease inhibitors (serpins) were observed. The extrinsic inhibition of serine protease activity *in vivo* resulted in distorted granuloma structure, extensive hypoxia, and increased bacterial growth in this model (80).

Active TB can result from either early progression of a primary granuloma during early infection or reactivation of an established granuloma in a latently infected person. People can be infected during infancy, develop latent TB and progress to disease after aging or other immunosuppressive conditions such as HIV co-infection (140). During active disease, the bacterium induces the death of the infected macrophages through necrosis, is released from the macrophage and replicates extensively using the liquified nutritive rich milieu of the caseum (141, 142).

For cavities to form, Mtb products indeed induce the overexpression of host proteases, like cathepsins, which are secreted from monocyte-derived cells, neutrophils, and stromal cells. These proteases ultimately destroy the lung parenchyma, in particular the collagen constituent of the extracellular matrix (ECM). Specific cysteine cathepsins such as K, S, and V present higher collagenolytic and elastolytic activities compared to neutrophil elastase or metalloproteinase collagenases (53). Importantly, enzymes responsible for collagen destruction are emerging as key targets for adjunctive therapies to limit immunopathology in TB (143).

Elevated levels of cathepsin B were observed in the lungs of mice and rabbits following infection with Mtb as well as in plasma from acute TB patients, suggesting that there is an association between increased cathepsin B levels and active disease (77). In addition, although lung granulomas are rich in cathepsin G and D, instead of being enrolled in bacteria killing as stated before, they seem to be more involved with cell autolysis, with the digestion of ingested necrotic debris and, consequently, with the process of liquefaction of the caseum, the most adverse event in the pathogenesis of TB (36, 144, 145).

Cavitation in TB enables highly efficient person-to-person aerosol transmission. Cathepsin K was another protease up-regulated in a rabbit cavitary TB model. Furthermore transcriptomic analysis in human lung specimens indicated cathepsin K gene up-regulation and showed that the protein levels were increased in the serum of patients with TB (146).

Many studies have identified potential biomarkers that discriminate active TB from LTB, or that are predictive of which individuals will progress to active TB. Therefore increased levels of cathepsin B or K in lungs or in circulation may be used as biomarkers to predict the evolution to TB from LTB. We indeed identified miR-106b-5p that targets cathepsin S as a potential biomarker to distinguish between active and LTB. This miR was described downregulated in samples from latently infected individuals (147) while in our studies, miR-106b-5p is up-regulated using experimental conditions that mimic active TB (28).

Co-infection with HIV has a profound effect on the dynamics of granuloma, which dramatically decreases the host protective responses to TB (**Figure 1**). HIV infection is known to deplete CD4^+^ T-lymphocytes by apoptosis and is detrimental for macrophages activation leading to less nitric oxide (NO) synthase expression and TNF-α (148). All these are required to maintain granulomas. In addition, high concentrations of proinflammatory cytokines greatly enhance HIV replication and among co-infected patients HIV load is greater in inflamed lung segments and in pleural fluid compared to plasma (24). Increased viral load may lead to locally increased mononuclear cell loss. Consequently, the pathogenesis of TB is different in HIV co-infected individuals, resulting in the lack of granulomas or in complete cavitation and in a higher incidence of disseminated disease.

Microarray analysis of peripheral blood of patients who had recovered from extrapulmonary tuberculosis compared to those who had recovered from pulmonary tuberculosis revealed that cathepsin W was strongly differentially expressed. Thus, cathepsin W is a potential biomarker for extrapulmonar TB diagnosis, a condition often found during co-infection with HIV (149).

A meta-analysis of peripheral blood transcriptome in co-infected patients revealed as potential biomarker for diagnostic of TB in HIV patients, the serpin inhibitor of serine proteases *SERPING1* gene (150). *SERPING1* codes for a protein called C1 inhibitor that targets the complement fractions C1r and C1s being essential for regulation of activation of the complement and kinin generating systems. Curiously, recent reports have highlighted complement as a candidate biomarker for active TB disease including in the presence of HIV co-infection. C1q levels were found elevated in patients with active TB compared to LTB in four independent cohorts (151).

Cathepsin K was also revealed as biomarker to discriminate pulmonary TB from other inflammatory lung diseases, such as adenocarcinoma and sarcoidosis that often mimic TB (152).

## Conclusions

Cathepsins and their inhibitors are extremely relevant players during Mtb and HIV infection. They may drive pathogen killing through direct contact and digestion or indirectly by modulating the immune responses. Generally, cathepsins are found in high amounts in the extracellular environments during chronic inflammation/infection contributing to transmission and to the abnormal pathological inflammation that can compromise organ functionality. Contrary they are usually found downregulated and inhibited in infected cells or, instead, they do not reach the same niche where the pathogen survives. While there are no proposed therapies based on cathepsin manipulation in the context of TB and HIV infections they are here presented as potential targets for new drug candidates. Thus, agents that restore protease–antiprotease balance by targeting protease inhibitors and/or protease activities, appear important to control infection, transmission and excessive inflammatory responses.

This may be achieved by directly addressing cathepsin activity inhibition in the extracellular environment. The history of cathepsin drug discovery has in fact witnessed the development of pharmacological cathepsin inhibitors, especially for cathepsin K during inflammatory conditions, as for osteoporosis or arthritis (143). Likewise, cystatins may potentially be used for adjunctive therapies to limit immunopathology in TB. The strategy to target pathogens in intracellular niches should attend other requisites in order to increase the proteolytic activity of cathepsins impacting pathogen killing and provide better antigen presentation. In this context, saquinavir is a good new possible HDT, as is the potential modulation of miR-106b (28, 29). In both cases an increased proteolytic activity especially of cathepsin S prove their efficacy. Other strategies might work by silencing cystatins such as C and F, both inhibitors of cysteine cathepsins that are major players in the endocytic pathway. Their inclusion in drug delivery systems such as nanoparticles or liposomes targeting host receptors is a good strategy to concentrate potential candidates in the intracellular milieu.

Moreover, differential expression of cathepsins and/or their inhibitors in active TB, LTB or during HIV co-infection, revealed a few as potential biomarkers for diagnostics. Indeed, the identification of physiological substrates of cathepsins or their inhibitors represents another area that is expected to expand substantially in the future as disease biomarkers.

In conclusion this area of research will open new avenues for the design of innovative therapies and diagnostic interventions so urgently needed to control TB-HIV threat to humanity.

## Author Contributions

Conceptualization: EA. Writing EA and JA-P. Editing: EA, JA-P, and DP. Images: EA. Supervision and funding acquisition: EA. All authors contributed to the article and approved the submitted version.

## Funding

This study was supported by grants from National Foundation for Science, FCT Fundação para a Ciência e Tecnologia – Portugal, PTDC/SAU-INF/28182/2017 to EA.

## Conflict of Interest

The authors declare that the research was conducted in the absence of any commercial or financial relationships that could be construed as a potential conflict of interest.

## Publisher’s Note

All claims expressed in this article are solely those of the authors and do not necessarily represent those of their affiliated organizations, or those of the publisher, the editors and the reviewers. Any product that may be evaluated in this article, or claim that may be made by its manufacturer, is not guaranteed or endorsed by the publisher.
